# Influencing Factors of Thriving at Work of Operating Room Nurses: A Qualitative Study Based on Social Ecosystem Theory

**DOI:** 10.1155/jonm/5573288

**Published:** 2026-06-22

**Authors:** Ru Li, Wenjuan Ma, Yuan Hu, Yueshu Xu, Li Li

**Affiliations:** ^1^ School of Nursing, Xinjiang Medical University, Urumqi, 830000, Xinjiang, China, xjmu.edu.cn; ^2^ Operating Room, The First Affiliated Hospital of Xinjiang Medical University, Urumqi, 830011, Xinjiang, China, xjmu.edu.cn; ^3^ Regional Population Disease and Health Care Research Center, Urumqi, 830011, Xinjiang, China

**Keywords:** operating room nurses, qualitative study, social ecosystem theory, thriving, thriving at work

## Abstract

**Background:**

The thriving at work of operating room nurses is crucial to patient safety, the quality of nursing care, and team stability. However, the current understanding of the status quo, influencing factors, and mechanisms underlying thriving at work among Chinese operating room nurses remains limited.

**Aim:**

This study aimed to explore the factors influencing thriving at work among operating room nurses based on social ecosystem theory.

**Design:**

A descriptive qualitative research design was used.

**Methods:**

Sixteen operating room nurses were recruited in Xinjiang from February to March 2025 and interviewed face‐to‐face. Data were analyzed using NVivo 12.0 software, and themes were identified via Colaizzi’s seven‐step analysis.

**Results:**

The analysis yielded three themes and nine subthemes aligned with the domains of social ecosystem theory: (1) microsystem: coping ability and personal character; (2) mezzosystem: work‐family role conflict and responsibilities, collaboration of the work team, positive feedback for work, colleague‐related emotional experiences, and nurse manager support and empowerment; and (3) macrosystem: societal perceptions of the nursing role and professional standards for operating room practice.

**Conclusions:**

From the perspective of social ecosystem theory, this study systematically identified influencing factors of operating room nurses’ thriving at work at micro‐, mezzo‐, and macrolevels for the first time. In the future, managers should attach importance to these factors, establish an individual‐organization‐society multidimensional support system, and implement personalized management strategies to facilitate coordinated development of individuals and organizations.

**Summary**
• Implications for the profession and/or patient care
◦ This study suggests that future measures to promote operating room nurses’ thriving at work should take factors within three interconnected systems into consideration. The results highlight the value of formulating targeted interventions guided by social ecosystem theory.
• Impact
◦ This study analyzes the factors affecting operating room nurses’ thriving at work and verifies the applicability of social ecosystem theory in exploring nurses’ real work experience. The findings can serve as a reference for formulating management strategies to enhance nurses’ thriving at work.
• Reporting method
◦ The results of the study were reported in accordance with the reporting guidelines of the Consolidated Standards for Reporting of Qualitative Research (COREQ).
• Patient or public contribution
◦ There were no contributions from patients and the public.

## 1. Introduction

The operating room (OR) is a specialized nursing unit where acute and critical patients receive surgical procedures. Its environment and nursing techniques differ from those of general wards [1], given the high‐risk, high‐stress, and highly collaborative nature of surgical work. OR nurses are responsible for preoperative visits, surgical supply preparation, intraoperative cooperation, postoperative environment arrangement, instrument management, and emergency rescue. They endure heavy physical labor and intense mental workload, accompanied by frequent emergency treatment and irregular rest time. Affected by the severe shortage of nursing staff, on‐the‐job nurses bear excessive workload and pressure and experience long‐term acute and chronic physical and psychological stress. Their physical and mental health is severely impaired, and they are susceptible to physical discomfort and reduced job satisfaction [2]. These problems not only weaken OR nurses’ work vitality but also adversely affect nursing quality and patient safety. To improve nursing quality and stabilize the nursing team, nursing managers should attach great importance to exploring ways to motivate OR nurses, maintain their work energy and efficiency, enhance job satisfaction, and promote personal professional growth and organizational performance.

## 2. Background

Advancements in medical technology require OR nurses to continuously update their professional knowledge of new surgical equipment and techniques to achieve effective teamwork, while standardizing nursing practices to ensure that technological innovations contribute to improved surgical outcomes and patient prognosis. This places high demands on the comprehensive competencies and professional skills of OR nurses [3], whose occupational pressure ranks among the highest across all clinical departments [4]. Long‐term persistent stress frequently induces occupational fatigue [5] and emotional exhaustion [6], which further deteriorates nursing quality and hinders the overall efficiency of surgical teams [7]. Therefore, in‐depth exploration of the factors influencing OR nurses’ thriving at work and the formulation of targeted intervention measures are urgently required to optimize nurses’ work experience and improve the overall quality of OR nursing services.

With the rise of positive psychology, organizational behavior, and other related disciplines, thriving at work, as a vital positive psychological state, has attracted widespread academic attention. It is defined as “the shared experience of vitality and learning”, referring to a psychological state in which employees simultaneously experience vitality and continuous learning at work [8]. Thriving at work provides both psychological and behavioral benefits: it regulates individual psychological status, promotes goal‐directed work behaviors, and improves individuals’ ability to adapt to the work environment and achieve occupational goals. Accordingly, it not only facilitates employees’ physical and mental health and personal development but also improves organizational effectiveness and promotes organizational prosperity [9]. Existing studies have confirmed that a higher level of thriving at work can enhance OR nurses’ constructive work behaviors, stimulate innovative motivation, increase job satisfaction, and reduce turnover intention [10].

Given its strengths in explaining person–environment interactions, social ecosystem theory, which was proposed by Bronfenbrenner [11], provides a suitable theoretical framework for understanding nurses’ thriving at work. This theory categorizes the social environments where individuals reside into three hierarchical systems: micro‐, mezzo‐, and macrosystems. The microsystem refers to the immediate social subsystem with which individuals directly interact; the mezzosystem is shaped by the interactions among proximal subsystems (e.g., families and workplace teams); and the macrosystem encompasses the broader cultural, institutional, and contextual backgrounds that govern these interactive processes within various micro and mezzo subsystems [12]. By systematically analyzing multilevel interactions between individuals and their surrounding environments, this theory has been widely and effectively applied to uncover individuals’ authentic psychological experiences and optimize clinical intervention strategies [13]. Accordingly, this study adopted social ecosystem theory to systematically explore the multilayered factors influencing OR nurses’ thriving at work across micro, mezzo, and macro dimensions.

Accordingly, this study adopts social ecosystem theory to systematically explore the micro‐, mezzo‐, and macrolevel factors influencing thriving at work among OR nurses. The findings can provide an empirical basis for nursing managers to develop targeted, personalized management strategies and further improve OR nurses’ thriving at work.

## 3. Research

### 3.1. Theoretical Framework

This study adopted Bronfenbrenner’s social ecosystem theory [11] to guide the development of the interview outline, thematic extraction, and result interpretation. Social ecosystem theory highlights the dynamic interactions between individuals and their social environments, classifying the human social ecological system into three core levels: the microsystem, the mezzosystem, and the macrosystem. Specifically, the microsystem comprises individual biological, psychological, and social subsystems; the mezzosystem includes small social units such as families and workplace teams; and the macrosystem covers large‐scale contextual environments involving organizations, communities, and social cultures [13].

Notably, the original ecological systems theory contains four hierarchical levels: microsystem, mesosystem, exosystem, and macrosystem. The present study simplified the theoretical framework into three dimensions (micro‐, mezzo‐, and macrosystems) based on two practical considerations. First, this study primarily focused on the most direct and salient environmental factors affecting the work experience of OR nurses. Second, existing qualitative nursing studies commonly integrate the exosystem into the macrosystem to enhance the framework’s adaptability to clinical practice. This three‐level simplified structure has been widely validated and applied in previous nursing research [14].

Benefiting from its multilevel analytical perspective, social ecosystem theory is well‐suited for exploring complex problems in nursing and healthcare research and has been extensively adopted in studies on caregiving experiences and health promotion. Previous studies regarding caregiver career adaptation [15] and patient illness experiences [14] have applied this theory to interpret multidimensional influencing factors, including individual psychological traits and stress coping at the microlevel, organizational environments and interpersonal interactions at the mezzolevel, and occupational training, professional learning, and value cognition at the macrolevel.

Nevertheless, few studies have comprehensively explored the multilevel factors associated with thriving at work among OR nurses from the perspective of social ecosystem theory. Accordingly, this theory provides a systematic and standardized theoretical framework for the present study to analyze the influencing factors of OR nurses’ thriving at work. At the microsystem level, this study focused on nurses’ personal attributes, exploring how individual psychological status and behavioral patterns fundamentally shape their work thriving. At the mezzosystem level, it deconstructed the workplace organizational environment and interpersonal interactions, analyzing both positive predictors (e.g., organizational support and team empowerment) and negative predictors (e.g., work‐family conflict and poor organizational climate), as well as their combined effects on nurses’ work thriving. At the macrosystem level, it further clarified the underlying impacts of social cultural contexts and professional value cognition on OR nurses’ thriving at work.

Furthermore, the three systems interact dynamically and mutually constrain one another, forming a complex multidimensional interactive network. Individual stress coping at the microlevel can affect mezzolevel team atmosphere and collaboration; insufficient mezzolevel organizational support further generates demands for macrolevel occupational training optimization and social professional recognition; and the macrolevel healthcare industry environment inversely shapes individual occupational experiences at the microlevel. This interactive mechanism enables a comprehensive and in‐depth understanding of the formation mechanism of OR nurses’ thriving at work, thereby providing solid theoretical evidence for developing targeted and precise intervention strategies in future nursing management.

### 3.2. Purpose of the Study and Research Questions

The purpose of this study was to explore the multilevel factors influencing thriving at work among Chinese OR nurses based on social ecosystem theory. This study addresses the following research question: What micro‐, mezzo‐, and macrolevel factors (encompassing individual attributes, team and organizational interactions, and sociocultural contexts) shape the thriving at work of OR nurses in China?

## 4. Methods

### 4.1. Research Design

This study adopted a descriptive qualitative research design to explore the factors influencing thriving at work among Chinese OR nurses. This approach is well‐suited for capturing the subjective work experiences and unquantifiable experiential demands of specific populations and has been widely applied in nursing and healthcare studies [16]. The present study was reported in accordance with the Consolidated Criteria for Reporting Qualitative Research (COREQ) guidelines.

### 4.2. Participants

Nurses in the OR were recruited through intentional sampling, and the study site was identified through convenience sampling. The study was conducted from February to March 2025 in the OR of a tertiary general hospital in Xinjiang, China. The sample size was determined based on the principle of information saturation. That is, interviews ended when no more new information or themes emerged [17].

Inclusion criteria were nurses engaged in OR work in tertiary hospitals with at least 2 years of working experience in the OR, good verbal expression and communication skills, and voluntary participation in the interview with a willingness to share genuine inner feelings and work experiences. Exclusion criteria included OR nurses who resumed work within 2 months after maternity leave, sick leave, or personal leave. These nurses were excluded because they remained in the post‐leave adjustment transition period, which may prevent them from providing stable and objective work experience descriptions and potentially introduce bias to qualitative data analysis.

### 4.3. Data Collection

Qualitative data were collected using a self‐developed semistructured interview outline. The preliminary outline was formulated based on literature review, the theoretical framework, and the clinical experience of the research team. The final version was revised and finalized according to expert consultation and preinterview results of three OR specialist nurses.

The interview outline covered the following core themes: (a) How do you perceive your learning initiative and work vitality in clinical practice? (b) What personal characteristics or habits help you cope with high work pressure and occupational challenges in the OR? (c) How do you balance work demands and family responsibilities, such as childcare and elderly care? (d) How do you experience interpersonal collaboration with team members, including surgeons and anesthesiologists? (e) How does feedback from colleagues and nurse managers influence your learning initiative and work vitality? (f) How has the implementation of industry standards and expert consensus affected your professional learning and work dynamism? (g) How does social recognition of the professional value of OR nurses influence your learning initiative and work vitality?.

One day prior to each interview, a suitable time and venue were confirmed with the participants. Before formal data collection, the researchers introduced the study purpose, methodology, and significance, clarified the connotation of thriving at work, and answered all questions raised by the participants. All interviews were conducted in an independent, quiet, and undisturbed conference room, with each interview lasting 40–60 min. During the interviews, participants’ facial expressions and body language were observed. All conversations were audio‐recorded and documented with field notes under the principles of voluntary participation and confidentiality. Flexible follow‐up questions were raised according to participants’ responses for in‐depth exploration.

Data saturation was confirmed when no new themes emerged from subsequent interviews. Upon completion of each interview, participants completed a general demographic questionnaire, and the researchers recorded field reflections in a timely manner. All audio recordings were transcribed verbatim by two researchers independently within 48 h after each interview.

### 4.4. Data Analysis

Colaizzi’s seven‐step thematic analysis was adopted for data coding and theme extraction. As a standardized analytical approach highly compatible with descriptive qualitative research, this method enables rigorous sorting, induction, and refinement of original interview data to objectively summarize participants’ authentic experiences, thereby ensuring the credibility and systematicity of qualitative findings [18]. Interview data were analyzed using NVivo 12.0 software (QSR International Pte Ltd). Combined with the methodological characteristics of descriptive qualitative research and the established theoretical framework.

The specific analytical procedures were as follows: (1) all interview recordings were transcribed verbatim and thoroughly reviewed within 24 h after each interview; (2) two researchers independently extracted meaningful statements closely related to nurses’ thriving at work; (3) initial coding was performed on the extracted meaningful statements; (4) codes were categorized and organized to form preliminary theme clusters; (5) the refined themes were comprehensively integrated and elaborated in accordance with the research context; (6) similar subthemes were summarized to clarify the structural framework and determine the major themes; and (7) the transcribed texts and preliminary results were returned to the participants for member checking.

The first two authors independently extracted themes and subthemes from the transcribed data. Any discrepancies in analysis were resolved through mutual discussion; unresolved disagreements were adjudicated by a third researcher in the research team.

### 4.5. Trustworthiness

The primary researcher is a master’s student who has received systematic training in qualitative research. All team members are proficient in qualitative research methodologies and techniques, ensuring the scientific and rigorous implementation of this study. Participant recruitment was undertaken by an OR head nurse to ensure efficient sample recruitment. Importantly, the recruitment coordinator did not participate in the one‐on‐one interviews to avoid interference. Throughout the interviewing process, the researcher remained neutral by conducting objective follow‐up inquiries without introducing personal biases or subjective opinions. To guarantee the credibility of the findings, ambiguous statements from participants were verified immediately during the interviews. All participants retained contact for subsequent follow‐up and supplementary inquiry. Two OR nurses provided additional supplementary information for the transcribed data, which was integrated into the final result interpretation.

### 4.6. Ethical Considerations

Written informed consent was obtained from all participants prior to data collection. All audio recordings and textual data containing personally identifiable information were fully anonymized to protect participant privacy. All research data were securely stored on a dedicated hard disk by the first author, with restricted access to unauthorized personnel. Participants were informed of their right to voluntarily withdraw from the study at any stage, with a guaranteed of no adverse impact on their daily work.

## 5. Findings

### 5.1. Characteristics of the Participants

In this study, data saturation was achieved at a sample size of 16 participants. The average age of the respondents was 32.69 years, with an average working experience of 8.94 years. Detailed demographic characteristics of the participants are presented in Table 1.

**TABLE 1:** General information of respondents (*n* = 16).

Participant number	Gender	Age (years)	Work experience (years)	Clinical ladder level	Marriage/childbearing	Academic qualifications
N1	Female	32	11	N2	Unmarried and childless	Bachelor’s degree
N2	Female	33	8	N2	Married with children	Bachelor’s degree
N3	Male	30	7	N2	Married with children	Specialized
N4	Male	39	16	N3	Married with children	Master’s degree
N5	Female	30	8	N2	Unmarried and childless	Bachelor’s degree
N6	Female	41	20	N4	Married with children	Bachelor’s degree
N7	Female	33	11	N3	Unmarried and childless	Master’s degree
N8	Male	28	4	N1	Unmarried and childless	Specialized
N9	Female	33	7	N2	Married and childless	Bachelor’s degree
N10	Female	28	6	N1	Unmarried and childless	Undergraduate
N11	Female	33	10	N3	Married with children	Bachelor’s degree
N12	Female	28	3	N1	Unmarried and childless	Specialized
N13	Female	32	7	N2	Married with children	Bachelor’s degree
N14	Female	33	14	N2	Married and childless	Undergraduate
N15	Female	35	8	N3	Married with children	Bachelor’s Degree
N16	Female	35	3	N0	Unmarried and childless	Specialized

*Note:* The “Clinical ladder level” (N0–N4) in this study follows the tiered nursing management system widely implemented in Chinese hospitals [19], guided by the National Health Commission of China. The classification is based on clinical experience, professional title, and competence: N0: New nurses with < 1 year of work experience. N1: Nurses in standardized training with 1–≤ 2 years of experience. N2: Registered nurses or nurse specialists with 2–≤ 5 years of experience. N3: Nurse specialists with 5–≤ 10 years of experience. N4: Supervisor‐level nurses with ≥ 10 years of experience. All participants are operating room nurses, not nurse practitioners (NPs) with independent prescriptive authority as defined in some Western jurisdictions. This multilevel sampling was adopted to ensure representation across different levels of clinical experience, enhancing the comprehensiveness of the qualitative findings.

### 5.2. Themes and Subthemes

Three themes and nine subthemes related to factors influencing thriving at work among OR nurses were identified, consistent with the domains of social ecosystem theory. The themes and representative quotes are presented in Table 2. The results are summarized as follows:

(a) Microsystem: coping ability and personal character.
(b) Mezzosystem: Work‐family role conflict and responsibilities, collaboration of work team, positive feedback for work, colleague‐related emotional experiences, and nurse manager support and empowerment.
(c) Macrosystem: societal perceptions of the nursing role and professional standards for or practices.

**TABLE 2:** Factors (themes) influencing nurses thriving at work.

System levels	Theme	Quote
Microsystem (individual)	Coping ability	“When it comes to work‐related stress, I mostly handle it on my own and rarely confide in friends because they can’t truly empathize and it only adds to their worries.” (N5)
	Personal Character	“During an 8‐h complex surgery, I tell myself, ‘Just hold on a little longer; the patient needs me more,’ and this belief helps me stay focused.” (N5)

Mezzosystem (groups)	Work‐family role conflict and responsibilities	“At home, my parents have to take care of the children. July and August are the peak surgery seasons, and I can’t see my children at all (shrugs). I start to question the meaning of work.” (N3)
	Collaboration of work team	“The operating room primarily operates under a multidisciplinary collaboration model, where a single surgery requires surgeons, anesthesiologists, instrument nurses, and circulating nurses. This collaborative work model makes me feel I’m not alone in this endeavor.” (N16)
	Positive feedback for work	“I have a thorough understanding of anatomical structures during surgery, and my collaboration with the surgeon is very smooth. He praises me, and I feel proud and honored. On such days, my work enthusiasm is very high.” (N10)
	Colleague‐related emotional experiences	“The attitudes of my colleagues also have an impact, especially those who don’t understand (the situation). For example, during this training program, some colleagues asked, ‘What’s the point of going for training? It’s better to just work here,’ which dampens my desire to learn.” (N12)
	Nurse manager support and empowerment	“I believe managers shouldn’t just focus on my nursing work. People are holistic beings. I hope the nurse manager can focus on cultivating our individual abilities. If certain tasks aren’t assigned to me, I can’t develop those skills.” (N10)

Macrosystem (external environment)	Societal perceptions of the nursing role	“New nurses start with enthusiasm, eager to save lives and heal the sick. However, upon encountering societal misconceptions—such as the notion that ‘nurses are merely doctors’ assistants with little technical expertise’—they may begin to doubt their career choice. Even minor challenges at work might lead them to question whether their field truly has a future. This significantly undermines their sense of professional belonging and commitment to their work.” (N8)
	Professional standards for OR practices	“The practicality of expert consensus is strong, and it can solve practical problems. For example, the detailed intraoperative pressure ulcer prevention steps and position diagrams have enabled us to successfully avoid pressure ulcers in major orthopedic surgeries. Such content that ‘can be applied immediately after learning and yields results when applied’ is more effective in stimulating learning initiative than pure theories. Now, the department often forwards and interprets it, and discusses how to apply it to surgical cooperation.” (N1)

### 5.3. Theme 1: Microsystem

#### 5.3.1. Coping Ability

In this study, many OR nurses reported that they generally tend to adopt the coping strategy of “internalizing emotions” when facing work pressure [20]. Although this strategy can alleviate emotional distress in the short term, long‐term emotional suppression not only impairs their physical and mental health but also leads to a vicious cycle of stress accumulation due to the lack of effective social support [21]. As stated by the following participant:

N6: “When I am in a very poor working condition and mood, I don′t really want to talk when I go home, so I go into my room and regulate myself.”

N5: “For work pressure, I basically regulate myself and rarely talk to my friends because they can’t really empathize and add to their worries.”

N7: “My physical examination results show that I have breast nodules, and my colleagues around me have such problems. I think it is caused by too much pressure, which is held in my heart without any way to resolve.”

N15: “I also don’t want to communicate with my family because they don’t work in healthcare and think that my current job is very good and think that I am making a big deal out of it.”

#### 5.3.2. Personal Character

Character strengths, defined as positive personality traits across multiple psychological dimensions including individual cognition, emotion, and behavior [22], play a unique protective role among OR nurses. Core traits such as resilience, curiosity, and responsibility constitute key psychological resources that enable these nurses to cope with professional challenges. These strengths not only mobilize positive emotions but also help nurses manage high‐intensity work pressure, pursue continuous professional growth, and maintain professional enthusiasm. The following participant quotes illustrate these points:

N5: “During a complex surgery that lasts for 8 h straight, I would tell myself ‘hold on just a little longer, the patient needs me more’, and this belief supports me to stay focused.”

N8: “When encountering unexpected intraoperative situations, my years of experience have taught me to respond calmly, and I believe that I am capable of handling every part of the process.”

N7: “When there is an academic conference on the weekend, I will definitely attend it as long as I am not on duty. Learning new techniques can help me cooperate with surgery more efficiently.”

N6: “I will regularly organize notes on surgical coordination and share them with newcomers. This habit not only helps others, but also consolidates my own professional ability.”

### 5.4. Theme 2: Mezzosystem

#### 5.4.1. Work‐Family Role Conflict and Responsibilities

Conflicts between the high workload of OR nursing and family responsibilities lead to role strain and family role absence among OR nurses, which in turn induces turnover intention and impairs work engagement.

N2: “Once my son was sick with fever and I was on the operating table, it was especially hard (sobbing) at that time, I felt that there was no way to take care of my child when he was sick, and I kind of didn’t want to do this job anymore.”

N3: “I can only rely on my parents to take care of my child at home, and when July and August are the peak months for surgeries, and I can’t see my child at all (on the table), I wonder what the point of the job really is.”

N4: “Sometimes when I go home and my child doesn’t do his homework, and when I criticize him, he will report to me and say that I don’t care about him all day long at work, and I am still here criticizing him. Sometimes it is very hard to hear such words in my heart, and I feel that I have too little time to accompany my child (sadness).”

#### 5.4.2. Collaboration of Work Team

The working environment in ORs differs from that in wards under the holistic nursing accountability system. Multidisciplinary teamwork fosters positive work emotions and enhances professional commitment among OR nurses.

N4: “In other words, it’s all about accountable care, and you’re relatively alone with your own independent division of patients, but in the operating room, there are at least two nurses, as well as the attending surgeon and the anesthesiologist, and I’ll be positively engaged in the surgery when we all work together.”

N16: “The operating room is mainly a multidisciplinary collaborative working model, an operation requires a surgeon, anesthesiologist, instrument nurse and a visiting nurse, and this working together makes me feel that I am not alone.”

N13: “An operating room is a team, when the surgeons and anesthesiologists are both very committed and efficient in their work, I definitely can’t afford to drop the ball and keep up with their pace as well, and actively engage in my work to successfully complete the surgical coordination.”

#### 5.4.3. Positive Feedback for Work

As a positive work emotion, a sense of accomplishment boosts job satisfaction among OR nurses and exerts positive effects on their work attitudes and behaviors.

N7: “We worked closely with the doctors to participate in the patient’s resuscitation, and by the time the resuscitation was successful, all of the clothes on the back were wet, and the sense of value and significance of the work was maximized, and this will have a great influence.”

N13: “When we finish our work or competitions with excellent performance, our leaders and colleagues will give us praise in the medical and nursing workgroup on WeChat, and I will feel very satisfied, and my work state will be more positive.”

N9: “When patients recovered and gave us flowers and banners, they felt that their efforts and dedication were seen and that their work was recognized by someone, and my working condition was very good in those days.”

N10: “I know the anatomy in the surgery very well, the cooperation with the surgeon is very smooth, he will praise me, I will feel proud and honored, and I will be very enthusiastic about my work on that day.”

#### 5.4.4. Colleague‐Related Emotional Experiences

As core members of the OR team, surgeons and nurses greatly influence OR nurses’ work status via their own attitudes and moods.

N11: “When the surgeon encounters an intraoperative hemorrhage, he himself gets irritated, everyone gets irritated, and the atmosphere is very poor, and my work status is definitely affected.”

N1: “If I am paired with a particularly temperamental doctor on the first day, and he is still him on the second day, I will be annoyed because some doctors are emotionally unstable, even if the operation goes well, and he encounters a slight mishap during the operation, he will keep rambling on and on, which is annoying, and it will affect my mood at work.”

N12: “The attitudes of our colleagues can also have an impact, especially the ones that don’t understand (attitudes), like this refresher study, some of our colleagues said what’s the use of going for refresher study? It’s better to work here, so that would dampen my desire to learn.”

#### 5.4.5. Nurse Manager Support and Empowerment

Failure of nursing managers to timely identify and develop nurses’ potential partly reduces nurses’ work engagement and enthusiasm for continuous learning.

N10: “I think the manager should not only care about my nursing work, people are a whole, I hope the nurse manager can focus on our individualized ability development, some tasks can’t be assigned to me, I have no way to exercise this aspect of my ability.”

N16: “The nurse manager did not value my strengths and potentials, and only focused on how well we can complete our nursing care.”

N15: “I hope the head nurse can give us more opportunities for development outside of nursing work, for example, I am good at editing videos and writing publicity scripts, but the people in charge of this work in the department are fixed, so I have no way to utilize my strengths, and I can only work as an OR nurse to cooperate with surgeries, which restricts my all‐round development.”

### 5.5. Theme 3: Macrosystem

#### 5.5.1. Societal Perceptions of the Nursing Role

Limited social recognition of the professional value of OR nurses adversely affects nurses’ professional mindset, work motivation, and other aspects in clinical practice.

N4: “For example, in emergency resuscitation surgery, the patient is hemorrhaging, we nurses have to quickly cooperate with the doctor, handing instruments and not making a mistake for a moment. But sometimes when we talk to our relatives and friends about our work, they think ‘it’s just handing a tool’, and they don’t even know the expertise and pressure we have to bear. This low social awareness makes us a little less motivated to work, as if we are doing a good job and the outside world doesn’t see your value.”

N8: “When new nurses first work, they are originally full of enthusiasm and think about saving lives. But after being exposed to some social misconceptions, such as some people think ‘nurses are just doctor’s assistants with little technical content’. They will slowly doubt their own choices, and when they encounter a little difficulty in their work, they may wonder if they really have no future. This is not a small blow to their sense of professional belonging and work commitment.”

#### 5.5.2. Professional Standards for OR Practices

The issuance and implementation of group standards and expert consensus exert dual effects on OR nurses’ learning motivation and work vitality. While they play a positive facilitating role, practical constraints may also bring certain limitations.

N1: “The expert consensus is highly practical and can solve practical problems, for example, the detailed steps for intraoperative pressure sore prevention and the positional schematic diagrams allowed us to successfully avoid pressure sores during major orthopedic surgeries, and this kind of content, which is “usable and effective after learning,” can stimulate the learning initiative more than pure theory. Now, the department often forwards it for interpretation and discusses how to apply it to surgical coordination.

N4: “In the past, different nurses had different habits when counting preoperative instruments, and sometimes they quarreled when the handover was not clear, which affected their mood and delayed their time. The new standard has clarified the counting requirements, so everyone follows it, and the surgical connection is smoother. I don’t have to worry about these trivial things anymore, instead I can focus on intraoperative cooperation, such as observing patients’ vital signs and passing instruments in time, and I feel that my work is more valuable and I don’t feel so tired when I’m busy.”

N11: “This year’s action plan includes the ‘Nurse Skills Enhancement Program’, which gives us a place to go to a higher‐level hospital for further training, and I signed up to learn nursing coordination for da Vinci surgery, which really broadened my horizons.”

## 6. Discussion

Through semistructured interviews, we found that OR nurses represent a high‐risk group for occupational fatigue [5], with most participants adopting an “internalizing emotions” coping style. Several key factors contribute to this prevalent coping tendency: excessive workload and chronically high‐stress occupational environments deplete nurses’ psychological resources [23]; nurses are reluctant to seek external psychological support; and they generally perceive that their unique occupational stress cannot be fully understood by others. Although this short‐term coping strategy can temporarily alleviate negative emotions and sustain basic work functioning, it inevitably triggers persistent adverse outcomes, including aggravated psychological burden and deteriorated physical and mental health [24]. These findings collectively indicate that OR nurses have substantial room for improvement in psychological adjustment and stress management abilities.

In contrast to the aforementioned passive coping status, some participants successfully overcame this occupational dilemma by leveraging their positive personal character strengths. Specifically, the psychological trait of resilience enables nurses to maintain mental toughness in high‐pressure clinical environments and serves as a fundamental psychological safeguard against chronic work stress. Meanwhile, curiosity and responsibility positively facilitate proactive learning behaviors, thereby improving overall work performance and sustaining work vitality.

These findings further reveal that character strengths exert dual regulatory effects among OR nurses with insufficient overall stress coping capabilities: they function as a critical psychological buffer against occupational stress and a core driving force for individual thriving at work. Accordingly, this study proposes the establishment of structured peer support intervention programs. Nurses with prominent character strengths can be selected and trained as workplace psychological coaches. Through systematic case presentations, regular group discussions, and standardized sharing of evidence‐based stress coping strategies, individual positive strengths can be effectively transformed into shared team psychological resources. This innovative strength transfer mechanism not only compensates for the deficiencies of traditional one‐way psychological support models but also fundamentally elevates the overall level of thriving at work within the nursing team.

From the mezzosystem dimension of the social ecosystem theory adopted in this study, teamwork and a sense of work accomplishment were identified as critical positive facilitators of thriving at work among OR nurses. Conversely, family role absence, negative emotional contagion from colleagues, and inadequate empowering leadership behaviors by head nurses constitute major barriers to nurses’ thriving at work. Among these inhibiting factors, family role absence was reported as the most prominent. Most participants stated that professional responsibility compels them to prioritize work demands over family needs, which induces intense familial guilt and even turnover intentions. These negative outcomes significantly reduce nurses’ job satisfaction and subjective well‐being [25]. Therefore, targeted organizational interventions are strongly recommended, including regular assessment of OR workload and working hours, rational adjustment of staffing age structure, and scientific human resource allocation [26]. These targeted measures can effectively enhance nurses’ self‐efficacy and perceived work–family support, alleviate work–family conflict, and further stabilize the OR nursing workforce.

In terms of mezzo‐level leadership factors, empowering practices of nurse leaders are recognized as a vital form of organizational support highly anticipated by OR nurses. Nurse empowerment refers to a standardized management approach, in which nurse managers grant nurses sufficient work autonomy to promote continuous professional competence development in clinical practice [27]. Nevertheless, participants reported that systematic empowering behaviors are generally insufficient in routine nursing management. Given the high‐risk nature of OR nursing work, preventing surgical adverse events and ensuring patient safety have always been the top clinical priorities. Under this rigorous work context, head nurses predominantly focus on solving nurses’ task‐based workplace problems. While they can timely identify clinical difficulties and provide targeted operational guidance, they often overlook the full exploration and cultivation of nurses’ potential capabilities. Consequently, nurses lack sufficient initiative to learn professional skills and improve their clinical competency.

Importantly, empowering leadership exhibits dual effects in clinical nursing management. Moderate and appropriate empowerment can effectively stimulate nurses’ self‐efficacy and intrinsic work motivation, thereby promoting proactive work behaviors and improving work engagement [28]. In contrast, excessive and blind empowerment forces nurses to expend extra time and energy to cope with challenging, overloaded work demands, resulting in sharply increased occupational pressure and psychological burden. For this reason, nursing managers are advised to precisely grasp the reasonable orientation and scale of empowerment implementation. Following the core principle of ability‐job matching, managers should allocate work tasks scientifically based on nurses’ individual characteristics and professional capabilities, to maximize the fit between organizational job resources and personal developmental needs, so as to effectively mobilize nurses’ work vitality and foster sustainable thriving at work.

In addition to leadership factors, organizational atmosphere and positive recognition also significantly influence nurses’ work status. Most interviewees confirmed that positive organizational recognition and a harmonious team atmosphere play indispensable roles in facilitating thriving at work. On the contrary, when individual team members display negative emotional states, the originally harmonious workplace atmosphere will become oppressive and tense, which exacerbates nurses’ risk of emotional exhaustion [29] and negatively impairs their occupational emotions. To optimize this situation, it is feasible to implement personalized recognition strategies and adopt the innovative appreciative inquiry model in nursing organizational management [30]. These strategies help build a positive organizational cultural atmosphere centered on affirmation and recognition and further promote nurses’ thriving at work. Meanwhile, hospitals should establish a sound emotional feedback mechanism to timely detect and curb the spread of negative work emotions among nurses so as to maintain a stable and harmonious organizational climate. Furthermore, nurses should strengthen self‐emotional regulation and personal self‐discipline, actively control the outward expression of negative emotions, and avoid negative emotional contagion within the nursing team.

At the macrosystem level, industry specifications also exert a profound impact on nurses’ professional development. The issuance and implementation of OR nursing group standards and expert consensus provide scientific theoretical guidance and a practical operational basis for standardizing nursing procedures, improving overall nursing quality, ensuring patient safety, and promoting the sustainable professional development of OR nurses [31], thereby positively driving the long‐term professional growth of nursing staff. On the one hand, expert consensus features high clinical pertinence and practicality, targeting practical problems in daily clinical work. The tangible benefits of integrating theoretical learning with clinical application stimulate nurses’ proactive learning enthusiasm, foster a departmental atmosphere of active knowledge sharing and academic communication, and transform nurses’ learning mode from passive acceptance to active exploration [32]. On the other hand, standardized group standards unify clinical workflows, reduce internal conflicts and work friction caused by nonstandard operations, and free nurses from cumbersome trivial work. This enables nurses to focus more on core intraoperative collaborative work, enhances their sense of work control and professional value, and sustains positive work vitality. Even in highly busy clinical scenarios, nurses can maintain positive occupational experiences, realizing the coordinated development of individual professional growth and continuous nursing quality improvement.

However, the implementation of industry standards also has potential limitations. Excessively intensive learning and training arrangements that conflict with the tight operational rhythm of OR work may weaken nurses’ enthusiasm for in‐depth professional learning [33] and reduce work vitality due to accumulated mental fatigue. Therefore, managers should formulate training plans in accordance with actual clinical work conditions and rationally adjust training timing and frequency to avoid occupying nurses’ rest time or overlapping with clinical peak periods. A phased and batch‐based training model is recommended to help nurses efficiently master professional knowledge and skills under a moderate workload. This model ensures training effectiveness without imposing excessive work burden, thereby facilitating the standardized and sustainable implementation of industry standards and expert consensus.

Beyond workplace micro and mezzo factors, macro social environmental factors also restrict nurses’ professional thriving. OR nurses generally suffer from insufficient societal recognition of their professional value. This dilemma is mainly attributed to the closed working environment of ORs and the lack of effective professional value publicity and communication channels, leading to inadequate public understanding and recognition of the professional connotation and core value of OR nursing work. Insufficient social recognition significantly undermines nurses’ professional identity, reduces their work enthusiasm and organizational commitment [15], and inhibits their initiative for autonomous learning, ultimately hindering their sustainable professional growth. Accordingly, a multidimensional professional value dissemination system should be constructed. By building professional science popularization platforms and diversified publicity channels, the unique professional value of OR nurses can be fully demonstrated. This helps shape a social atmosphere that respects the nursing profession, improve public recognition of OR nursing work, enhance nurses’ professional identity, and stimulate their motivation for learning and innovation so as to effectively promote long‐term thriving at work among OR nurses.

## 7. Limitations

This study has several limitations. First, participants were recruited from only one tertiary general hospital in Xinjiang, China, which may cause potential sample selection bias. Future studies are recommended to conduct multicenter or comparative research on the support needs for thriving at work among OR nurses so as to provide more targeted intervention strategies for diverse nurse groups. Second, the one‐time cross‐sectional interviews failed to capture the dynamic changes in OR nurses’ needs for thriving at work. Longitudinal follow‐up interviews are suggested to obtain more in‐depth and valuable evidence in future investigations.

## 8. Conclusion

Guided by social ecosystem theory, this qualitative study explored the multidimensional factors influencing thriving at work among OR nurses. The results indicate that OR nurses’ thriving at work is jointly shaped by micro‐, mezzo‐, and macrosystemic factors. At the microlevel, personal character and coping ability influence OR nurses’ thriving at work. At the mezzolevel, key factors include work‐family role conflict and responsibilities, collaboration of the work team, positive feedback for work, colleague‐related emotional experiences, and nurse manager support and empowerment. At the macrolevel, societal perceptions of the nursing role and professional standards for OR practices jointly shape nurses’ thriving at work. The social ecosystem theory comprehensively explains these complex influencing mechanisms and provides a solid theoretical basis for targeted interventions. Future multisystem strategies focusing on improving nurses’ stress coping capacity, optimizing organizational management and team climate, implementing stratified training, and enhancing social professional recognition can effectively support and promote OR nurses’ thriving at work.

### 8.1. Implications for Future Research and Practice

Based on the findings of this study, we propose several directions for future research and practice. Drawing on the social ecosystem theory analysis of factors influencing thriving at work among OR nurses, we offer the following implications for clinical practice and research:

First, targeted interventions should be implemented to enhance nurses’ stress management abilities through systematic training. Special attention should also be paid to exploring and reinforcing nurses’ personal character strengths to strengthen their intrinsic resilience when facing professional challenges.

Second, it is recommended to optimize the organizational environment and interpersonal interactions in the OR. Efforts should include improving the organizational support system to empower nurses, establishing efficient collaborative platforms to promote teamwork, and enhancing nurses’ sense of accomplishment through clear career development pathways and formal recognition mechanisms. Meanwhile, measures should be taken to alleviate work‐family conflicts, improve the detached organizational climate, and strengthen the empowering capacity of nurse managers to build a supportive middle‐management model.

In addition, to address macrolevel issues related to professional training and public value perceptions, the positive role of learning group standards and expert consensus should be further leveraged while exploring practical solutions to their existing limitations. Publicity on the social value of the OR nursing profession should also be intensified to mitigate insufficient societal recognition of nurses’ professional worth.

Future research could explore the underlying mechanisms through which macrosystemic factors influence nurses’ thriving at work, thereby providing more robust theoretical evidence for policy‐making. This study also highlights the need to build a synergistic multisystem support network spanning micro‐, mezzo‐, and macrolevels to comprehensively promote thriving at work among OR nurses.

## Author Contributions

Conceptualization, study design, semistructured interview data collection, thematic analysis, data interpretation, original draft preparation, and manuscript revision: Ru Li; participant recruitment support, data transcription, formal analysis, and manuscript revision: Wenjuan Ma; participant recruitment support, member checking of interview transcripts, supplementary data verification, and manuscript revision: Yuan Hu; data curation, methodological consultation, and manuscript revision: Yueshu Xu; methodology supervision, study design guidance, critical revision of the manuscript for important intellectual content, funding acquisition, project administration, and final approval of the version to be submitted: Li Li.

## Funding

This study was supported by the 2024 Xinjiang Uygur Autonomous Region “Tianshan Yingcai” High‐level Talent Cultivation Program for Medicine and Health (Grant no. TSYC202401A075).

## Disclosure

All authors read and approved the final version of the manuscript.

## Ethics Statement

This study was approved by the Ethics Committee of the First Affiliated Hospital of Xinjiang Medical University (Approval No. K202412‐10; December 10, 2024).

## Consent

Written informed consent was obtained from all participants prior to data collection.

## Conflicts of Interest

The authors declare no conflicts of interest.

## Data Availability

Data are available on request from the authors.
